# Complement C3a promotes proliferation, migration and stemness in cutaneous squamous cell carcinoma

**DOI:** 10.1111/jcmm.13959

**Published:** 2019-03-01

**Authors:** Zhuo Fan, Jingjing Qin, Dandan Wang, Songmei Geng

**Affiliations:** ^1^ Department of Dermatology The Second Affiliated Hospital of Xi'an Jiaotong University Xi'an China; ^2^ Department of Dermatology The First Affiliated Hospital of Xi'an Medical University Xi'an China

**Keywords:** complement C3a, cutaneous squamous cell carcinoma, migration, proliferation, stemness

## Abstract

**Background:**

Complement C3 has been shown to be highly expressed in cutaneous squamous cell carcinoma (cSCC) tumour tissues and is correlated with tumour cell growth. This study aimed to investigate the mechanism of C3 in cSCC malignant transformation.

**Methods:**

C3 expression was analysed in cSCC cell lines A431, Tca8113, SCC13, HSC‐5 and HSC‐1 and in immortalized HaCaT keratinocytes. Proliferation and migration of cSCC were determined after C3a exposure. Expression of cyclin D1, cyclin E, vascular endothelial growth factor (VEGF), pro‐matrix metalloproteinase 1 (pro‐MMP1), pro‐matrix metalloproteinase 2 (pro‐MMP2), stemness factors, GSK‐3β, and β‐catenin were analyzed. Tumour growth was examined in a murine xenograft model.

**Results:**

C3 expression was much more highly expressed in all cSCC cell lines than in HaCaT cells. C3a treatment significantly promoted cSCC cell proliferation and migration and upregulated cyclin D1, cyclin E, VEGF, pro‐MMP1 and pro‐MMP2 expression, which were impeded by the C3aR antagonist. Moreover, the expression of stemness factors Sox‐2, Nanog, Oct‐4, c‐Myc and CD‐44 was stimulated by C3a and slowed by C3aR disruption. Knockdown of Sox‐2 by siRNA transfection suppressed cell proliferation and migration, constrained VEGF secretion and inhibited pro‐MMP1 and pro‐MMP2 expression. C3a also activated the Wnt and β‐catenin pathway in cSCC cells. Disruption of C3aR expression dampened tumour growth and the expression of Wnt‐1, β‐catenin and Sox‐2 in the xenograft model.

**Conclusions:**

C3a enhanced cell proliferation, migration and stemness in cSCC, and this activity was correlated with activation of the Wnt and β‐catenin pathway.

## 1 INTRODUCTION

Cutaneous squamous cell carcinoma (cSCC) is the second‐most common nonmelanoma skin cancer, accounting for nearly 20% of such cancers in the United States.^1^ It is most common in Caucasian ethnic groups.^2^, ^3^ This malignant skin disease is associated with high morbidity and mortality. Major risk factors for cSCC development include prolonged ultraviolet exposure and immunosuppression associated with human papillomaviruses.^4^, ^5^, ^6^

Inflammatory processes and factors are activated in cSCC tissues and pathogenesis.^7^ The complement system is a critical part of innate immunity against pathogen invasion. This system activates through the classical, alternative and lectin pathways, during which a cascade of enzymatic reactions stimulate multiple proteins.^8^ Recently, the role of the complement pathway in cancer growth has been elucidated. Complement anaphylatoxin (C3a), which is the active form of C3, and C3a receptor signalling could promote melanoma development and growth.^9^ Binding of C3a with its receptor negatively regulates E‐cadherin expression, which promotes the invasive phenotype in tumour cells.^10^ Other complement factors have been implicated in lung,^11^ breast,^12^ colon^13^ and pancreatic cancers.^14^ In cSCC tissues, complement factor H expression increased the growth and migration advantage of cSCC cell lines, whereas silencing complement factor H expression reduced this growth and migration.^15^ C3 expression was upregulated more in primary and metastatic CSCC cells than in normal epidermal keratinocytes.^15^ Nevertheless, the role of C3 in cSCC remains unknown.

Sex determining region Y‐Box 2 (Sox2) is a member of the SOX family. It contains a high‐mobility domain, which can bind specifically to a DNA sequence and regulate downstream gene expression. Sox2 maintains cell stemness and is essential in induced pluripotent stem cells.^16^ Alterations in Sox2 expression cause developmental diseases,^17^ and amplification of Sox2 occurs in many cancers. High expression of Sox2 is critical for maintaining cancer stem cells.^18^ Ectopic Sox2 expression also reduces tamoxifen sensitivity in cancer cells.^19^ Several factors regulate Sox2 expression at the transcriptional level in mouse and human cSCC. Deletion of Sox2 causes cSCC tumour regression and malignant transformation.^20^ Sox2 expression regulates the Nrp1 and vascular endothelial growth factor (VEGF) pathway, which causes cSCC proliferation by facilitating tumour‐initiating cells to generate more undifferentiated tumour cells.^21^ The current study sought to explore the role of C3a in cSCC and its association with Sox2. The results indicate that the complement system plays a role in cSCC carcinogenesis and thus is a potential target for tumour therapy.

## 2 MATERIALS AND METHODS

### 2.1 Cutaneous squamous cell carcinoma culture and treatment

The cSCC cell lines HSC‐1 and HSC‐5 were obtained from the Japanese Collection of Research Bioresources Cell Bank (Osaka, Japan). A431 cells were obtained from the American Type Culture Collection (Manassas, VA, USA). SCC13 cells were kindly provided by Prof. Paolo Dotto of the Cutaneous Biology Research Center at Massachusetts General Hospital in Charlestown, MA, USA. Tca8113 cells were purchased from the China Center for Type Culture Collection (Wuhan, China). Cells were cultured in Dulbecco's modified Eagle's medium supplemented with 10% fetal bovine serum, penicillin (100 U/mL) and streptomycin (100 μg/mL; Invitrogen, Carlsbad, CA, USA). Cells were grown under a humidified atmosphere of 5% CO_2_ at 37°C.

A431 and SCC13 cells were exposed to a human C3a peptide agonist, as described in a previous study.^22^ A431 and SCC13 cells were treated with 0.1 and 0.2 μmol/L of C3a respectively, for 24 hours. To block C3aR activity, A431 and SCC13 cells were pretreated with the C3aR antagonist SB290157 (0.2 μmol/L) for 24 hours. The control group was treated with an equivalent volume of saline.

### 2.2 The siRNA and plasmid transfection

Duplex siRNAs targeting human C3aR (NM_004054.3) and Sox‐2 (KR091848.1) were designed and synthesized by GenePharma (Shanghai, China). Scramble siRNAs were used as control siRNA (random siRNA duplex). DKK‐1 mRNA was reverse‐transcribed into cDNA and subcloned into the mammalian expression vector pcDNA3.1 (Invitrogen). A431 and SCC13 cells (5 × 10^5^ cells per well) were seeded in six‐well plates and grown to 70%‐90% confluence. The siRNA and plasmid transfections were carried out with Lipofectamine 2000 (Invitrogen) according to the manufacturer's protocol. Briefly, Lipofectamine 2000 (12 μL) was diluted into 150 μL of Opti‐MEM medium. Then, 14 μg of plasmids or 30 pmol of siRNAs were diluted into 700 μL of Opti‐MEM medium and mixed with 150 μL of diluted Lipofectamine 2000 at a 1:1 ratio. The mixture was then incubated for 5 minutes at room temperature. The plasmid mixture or siRNA mixture was then added to the cells, which were incubated for 48 hours in a 5% CO_2_ atmosphere at 37°C. After the transfection, western blotting was used to examine the transfection efficacy.

### 2.3 Cell viability

A431 and SCC13 cell viability was evaluated using the 3‐(4,5‐dimethylthiazol‐2‐yl)‐2,5‐diphenyl‐2H‐tetrazolium bromide assay. Cells were placed in 96‐well plates (1 × 10^5^ cells per mL) and treated as indicated. The medium was then removed and changed into normal Eagle's MEM with 10% FBS for another 24‐hours culturing. The determination of cell viability was carried out according to the manufacturer's protocols (#ST316; Beyotime, Jiangsu, China). After incubation for 4 hours, DMSO (150 μL per well) was added to end the reaction and the absorbance was determined in an ELISA plate reader at 570 nm at the indicated time.

### 2.4 Cell migration

A431 and SCC13 cell (3 × 10^5^/well) were plated in 100 μL serum‐free DMEM in the upper chambers of Transwell™polycarbonate inserts (Corning Costar, NY, USA) with 8 μm pores. In the bottom chamber, DMEM with 10% fetal calf serum was added. After incubation for 4 hours, cells on the lower side of the filter were fixed, stained and counted.

### 2.5 Enzyme‐linked immunosorbent assay

A431 and SCC13 cells (3 × 10^5^ cells per well) were seeded into six‐well plates and treated for 24 hours with 0.1 and 0.2 μmol/L of C3a, respectively, and with C3aR siRNA and Sox‐2 siRNA or the control siRNA transfection. After that, cell supernatants were collected and centrifuged to remove cell debris. VEGF concentrations in each treatment were measured using a human VEGF ELISA Kit (R&D Systems, Minneapolis, MN, USA) according to the protocol instructions. Pro‐MMP1 and pro‐MMP2 concentrations were measured by human pro‐MMP1 and pro‐MMP2 ELISA Kit (R&D Systems) respectively.

### 2.6 Tumour xenograft model

A431 and SCC13 cells were transfected with C3aR siRNA or control siRNA. After the stable transfection, tumour cells (5 × 10^6^) were suspended in 100 μL of phosphate‐buffered saline and subcutaneously injected into the back of severe combined immunodeficiency (SCID/SCID) female mice (5‐6 mice per group). Body weights and tumour sizes of mice were measured every week. Tumour volume was calculated using the formula *V* = (length × width^2^)/2. At the fourth week after injection, tumours were harvested for further analysis.

### 2.7 Real‐time quantitative PCR

The cSCC cells or frozen homogenized tumour tissues and total RNA were extracted with an RNeasy kit (Qiagen, Hilden, Germany) according to manufacturer instructions. RNA concentrations were detected with a Nanodrop 2000 (ThermoFisher Scientific, Shanghai, China) and purified with an RNA purification kit (Tiangen, Beijing, China). The first strand of cDNA was synthesized using a OneStep RT‐PCR Kit (Qiagen). Quantitative PCR then was conducted using a SYBR Green PremixEx Taq (Takara, Dalian, China). Primers used in the reactions were listed in Table 1. Data were analysed via the delta/delta cycle threshold (CT) method.

**Table 1:** Primers used in qRT‐PCR reaction

Gene symbols	Sequence (5′‐3′)
C3	Forward: AGATAAGAACCGCTGGGAGG
	Reverse: CACGACGGGAGGCACAAA
Sox‐2	Forward: ATGGGTTCGGTGGTCAAGTC
	Reverse: CGCTCTGGTAGTGCTGGGACA
Nanog	Forward: CACCTATGCCTGTGATTT
	Reverse: CAGAAGTGGGTTGTTTGC
Oct‐4	Forward: AAGGGCAAGCGATCAAGC
	Reverse: GGAAAGGGACCGAGGAGTA
c‐Myc	Forward: CATCCTGTCCGTCCAAGC
	Reverse: ACGCACAAGAGTTCCGTAG
CD‐44	Forward: CATAGAAGGGCATGTGGT
	Reverse: GTGTCATACTGGGAGGTGT
β‐actin	Forward: CGGGAAATCGTGCGTGAC
	Reverse: CAGGCAGCTCGTAGCTCTT

### 2.8 Western blotting

The cSCC cells or frozen tumour tissues were lysed by RIPA lysis buffer. Proteins were extracted, measured and analysed by western blotting. Briefly, 40 μg per lane of protein was separated by 10% (w/v) sodium dodecyl sulfate polyacrylamide gel electrophoresis and then transferred onto a polyvinylidene fluoride membrane. Then, 5% (w/v) bovine serum albumin (BSA) was used to block nonspecific binding in the membrane. After washing with Tris buffered saline with Tween buffer, membranes were incubated overnight at 4°C with primary antibodies. Primary antibodies included anti‐C3 (1:800; Abcam, Cambridge, MA, USA), anti‐cyclin D1 (1:800; Abcam), anti‐cyclin E1 (1:800; Abcam), anti‐VEGF (1:800; Abcam), anti‐pro‐MMP1 (1:800; Abcam), anti‐pro‐MMP2 (1:800; Abcam), anti‐Sox‐2 (1:800; Abcam) and anti‐DKK‐1 (1:800; Abcam). Horseradish‐peroxidase‐conjugated secondary antibody was then added, and cells were incubated for 2 hours at room temperature. Signals of target protein were visualized by incubating the membranes with chemiluminescence reagents and then quantifying with the ImageJ program (National Institutes of Health, Bethesda, MD, USA). Β‐actin was used as the loading control for normalization.

### 2.9 Immunohistochemistry analysis

Tumour tissues were fixed by formalin, embedded by paraffin and cut into sections (4 μm thickness). The sections were then deparaffinized, rehydrated, immersed in 3% hydrogen peroxide solution for 10 minutes, boiled in citrate buffer (pH 6.0) for 5 minutes and then cooled at room temperature for 2 hours. Following that, the sections were blocked with 5% BSA to reduce non‐specific antibody binding. Rabbit antibodies against β‐catenin, and Sox‐2 were then used to detect target protein expression (4°C overnight). The bound antibodies were then detected using the GTvision two‐step histostaining reagent (Genetech, Shanghai, China) and DAB (3,3′‐diaminobenzidine tetrahydrochloride) substrate.

### 2.10 Statistical analysis

Obtained data are expressed as mean ± SD. One‐way ANOVA was employed to compare the difference among multiple groups. Statistical software SPSS 13.0 (SPSS Inc., Chicago, IL, USA) was used for data analysis and comparison. *P*‐values less than 0.05 were considered significant.

## 3 RESULTS

### 3.1 C3 was highly expressed in tumour cell lines

**Figure 1:**
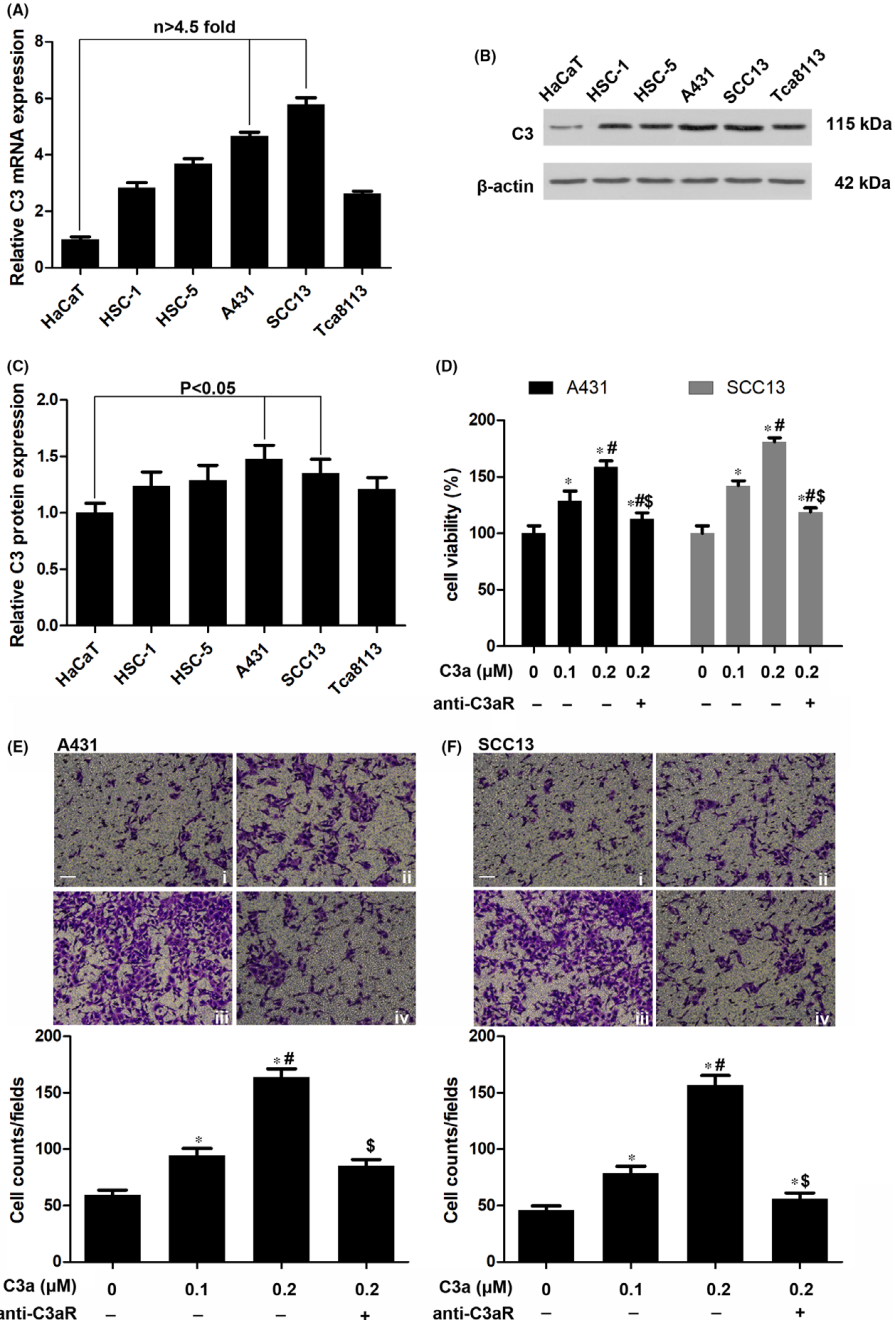
The expression of complement C3 in cutaneous squamous cell carcinoma (cSCC) cell lines. (A) Analysis of C3 mRNA in HaCaT, HSC‐1, HSC‐5, A431, SCC13 and Tca8113 cells by qRT‐PCR. n = 4. (B, C) Western blotting of C3 protein in cSCC cells. n = 4. (D) Evaluation of cell viabilities by MTT assay after the exposure of different concentrations (0.1 and 0.2 μmol/L)of C3a. n = 5. *P < 0.05 vs 0 μmol/L C3a, #*P* < 0.05 vs 0.1 μmol/L C3a, $*P* < 0.05 vs 0.2 μmol/L C3a. (E, F) Evaluation of cell migration by transwell asaay after C3a exposure. n = 4. i indicates 0 μmol/L C3a, ii indicates 0.1 μmol/L C3a, iii indicates 0.2 μmol/L C3a, iv indicates 0.2 μmol/L C3a+anti‐C3aR. Scale bar = 20 μm. *P<0.05 vs 0 μmol/L C3a, #*P*<0.05 vs 0.1 μmol/L C3a, $*P* < 0.05 vs 0.2 μmol/L C3a

We compared the expression of C3 mRNA in a series of cSCC cell lines, including HSC‐1, HSC‐5, A431, SCC13 and Tca8113, as well as in human immortalized HaCaT keratinocytes for the normal control. The results showed that C3 mRNA expression was upregulated in all tumour cell lines and was more than 4.5 times higher in A431 and SCC13 cells (Figure 1A). Western blotting showed that C3 protein expression at the translational level increased significantly more in cSCC cell lines than in HaCaT cells (Figure 1B,C). The results demonstrate increased C3 expression in several cSCC cell lines.

### 3.2 C3a modulated cutaneous squamous cell carcinoma proliferation and migration

**Figure 2:**
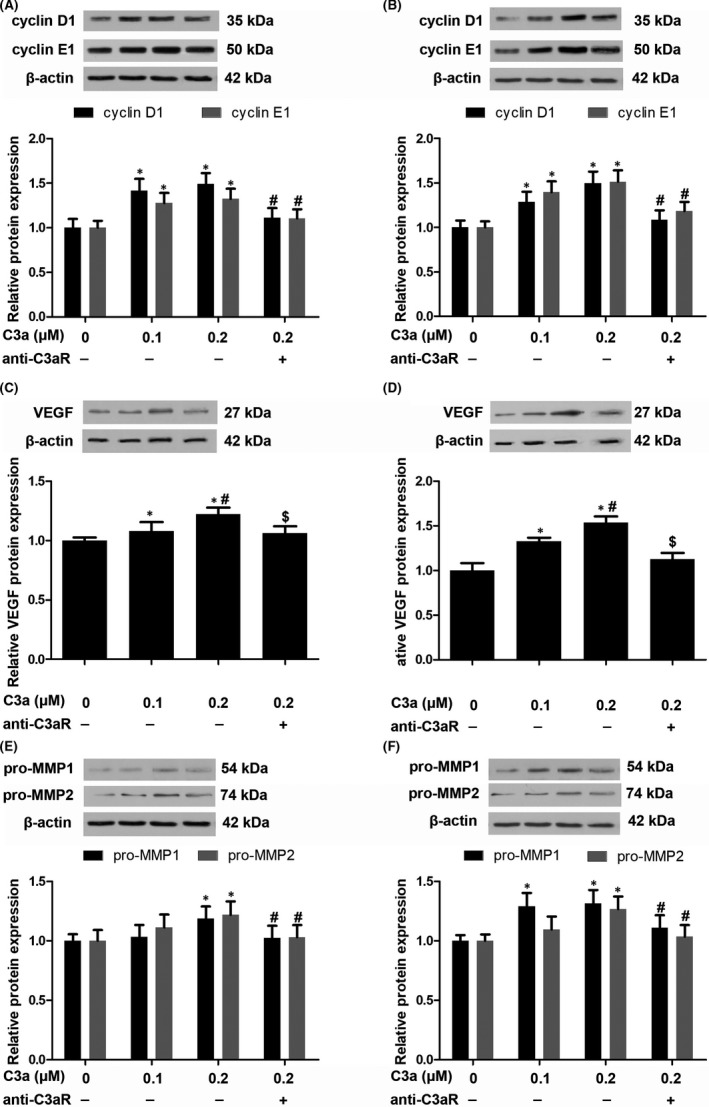
The effect of C3a on cutaneous squamous cell carcinoma (cSCC) cells. (A, B) Western blotting of cyclinD1 and cyclin E1 in A431 and SCC13 cells secondary to C3a exposure. n = 4. *P < 0.05 vs 0 μmol/L C3a, #*P* < 0.05 vs 0.2 μmol/L C3a. (C, D) Western blotting of VEGF in A431 and SCC13 cells. n = 4. *P < 0.05 vs 0 μmol/L C3a, #*P* < 0.05 vs 0.1 μmol/L C3a, $*P* < 0.05 vs 0.2 μmol/L C3a. (E, F) Western blotting of pro‐MMP1 and pro‐MMP2 in A431 and SCC13 cells. n = 4. *P < 0.05 vs 0 μmol/L C3a, #*P* < 0.05 vs 0.2 μmol/L C3a

High expression of the C3 protein in cSCC cell lines and in C3a releases C3 cleavage, which induces tumour growth via receptor C3aR. To explore the function of C3a in cSCC, A431 and SCC13 cells were treated with a C3a mimetic peptide. Our results showed that 0.1 and 0.2 μmol/L of C3a significantly increased the viability of A431 and SCC13 cells (Figure 1D). Rather than control, the C3aR antagonist abolished the effect of C3a on cSCC cell viability (Figure 1D). Results of the transwell assay showed that C3a treatment markedly promoted and that C3aR antagonist diminished cSCC tumour cell migration (Figure 1E,F). In parallel, C3a upregulated the expression of cyclin D1 and cyclin E in these cells. And cyclin expression was decreased by C3aR antagonist (Figure 2A,B).

### 3.3 C3a induced vascular endothelial growth factor and matrix metalloproteinase expression in cutaneous squamous cell carcinoma

Vascular endothelial growth factor is correlated with cancer lymph node metastasis due to its function in inducing angiogenesis. Complement factor deficiency has been linked to poor vascularization in ovarian cancer.^23^ Hence, we explored whether C3a strengthens the proangiogenic phenotype of cSCC cells. In the A431 cell line, 0.2 μmol/L of C3a increased cellular VEGF protein by about 1.2‐fold (Figure 2C). In the SCC13 cell line, 0.1 and 0.2 μmol/L of C3a increased VEGF protein by about 1.3‐ and 1.5‐fold respectively (Figure 2D). C3aR antagonist blocked the induction of VEGF expression, indicating that C3aR participates in C3a function (Figure 2C,D). Activation of matrix metalloproteinases contributed to extracellular matrix remodelling and facilitated cancer cell metastasis. We also observed elevated expression of pro‐matrix metalloproteinase 1 (pro‐MMP1) and pro‐matrix metalloproteinase 2 (pro‐MMP2) expression in cSCC cells that were exposed to C3a (Figure 2E,F). However, the C3aR antagonist decreased C3a‐induced expression of pro‐MMP1 and pro‐MMP2 (Figure 2E,F).

### 3.4 C3a treatment potentiated stemness factor expression in cutaneous squamous cell carcinoma

**Figure 3:**
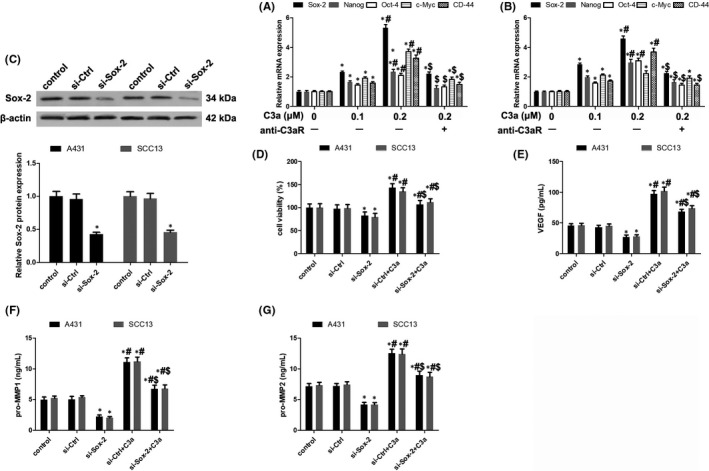
The involvement of Sox‐2 in C3a functioning. (A, B) Expression analysis of stemness factors including Sox‐2, Nanog, Oct‐4, c‐Myc and CD‐44 in A431 and SCC13 cells. n = 4. *P < 0.05 vs 0 μmol/L C3a, #*P* < 0.05 vs 0.1 μmol/L C3a, $*P* < 0.05 vs 0.2 μmol/L C3a. (C) Knockdown of Sox‐2 expression in A431 and SCC13 cells. n = 4. *P < 0.05 vs control. (D) Evaluation of cell viabilities after Sox‐2 knockdown with or without C3a exposure. n = 5. *P < 0.05 vs control, #*P* < 0.05 vs si‐Sox‐2, $*P* < 0.05 vs si‐Ctrl+C3a. (E) Detection of VEGF secretion by ELISA assay after Sox‐2 knockdown with or without C3a exposure. n = 5. *P < 0.05 vs control, #*P* < 0.05 vs si‐Sox‐2, $*P* < 0.05 vs si‐Ctrl+C3a. (F) Detection of pro‐MMP1 secretion by ELISA assay. n = 5. *P < 0.05 vs control, #*P* < 0.05 vs si‐Sox‐2, $P < 0.05 vs si‐Ctrl+C3a. (G) Detection of pro‐MMP2 secretion by ELISA assay. n = 5. *P < 0.05 vs control, #*P* < 0.05 vs si‐Sox‐2, $*P* < 0.05 vs si‐Ctrl+C3a

A previous study has linked complement factors with tumour growth and stemness in cancer cells.^24^ Given that C3a prompted cSCC cell proliferation and migration, we explored whether C3a influenced stemness factors Sox‐2, Nanog, Oct‐4, c‐Myc and CD‐44. In comparison with the control, C3a caused significant elevation of mRNA in Sox‐2, Nanog, Oct‐4, c‐Myc and CD‐44, which was lowered by C3aR blockade (Figure 3A,B).

To examine the functions of stemness factors in cSCC malignant transformation, we silenced the expression of Sox‐2, which is the most upregulated stemness factor (Figure 3A,B), by transfection with Sox‐2 siRNA (Figure 3C). The results showed that Sox‐2 disruption decreased cell viability induced by C3a (Figure 3D). Silencing Sox‐2 expression also constrained VEGF secretion and pro‐MMP1 and pro‐MMP2 expression in cSCC cells (Figure 3E‐G). These results indicate that C3a might potentiate stemness factors by promoting cSCC cell proliferation and migration.

### 3.5 C3a treatment activated Sox‐2 via the Wnt and β‐catenin pathway

**Figure 4:**
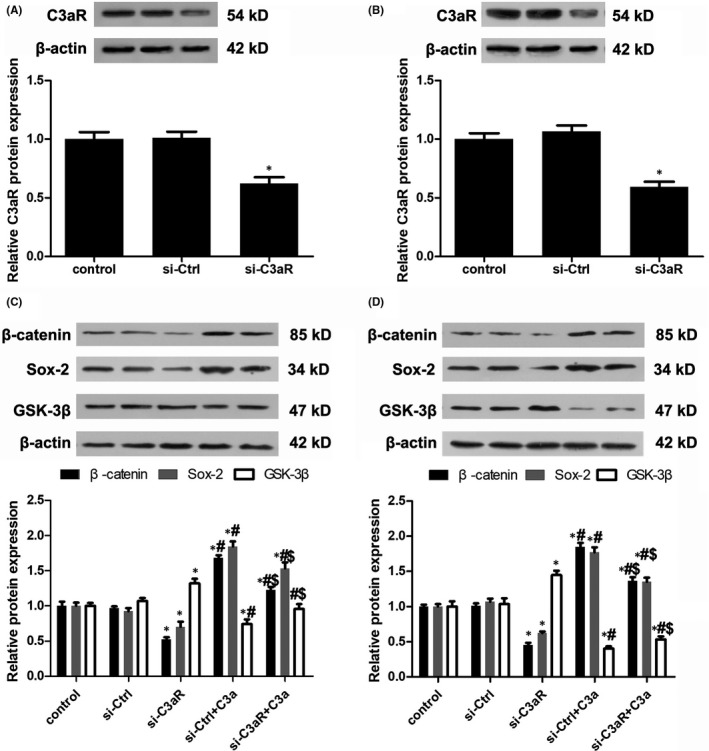
Activation of Wnt/β‐catenin pathway in C3a functioning. (A, B) Knockdown of C3aR expression in A431 and SCC13 cells. n = 4. *P < 0.05 vs control. (C, D) Analysis of β‐catenin, Sox‐2 and GSK‐3β expression in A431 and SCC13 cells. n = 4. *P < 0.05 vs control, #*P* < 0.05 vs si‐C3aR, $*P* < 0.05 vs si‐C3aR+C3a

**Figure 5:**
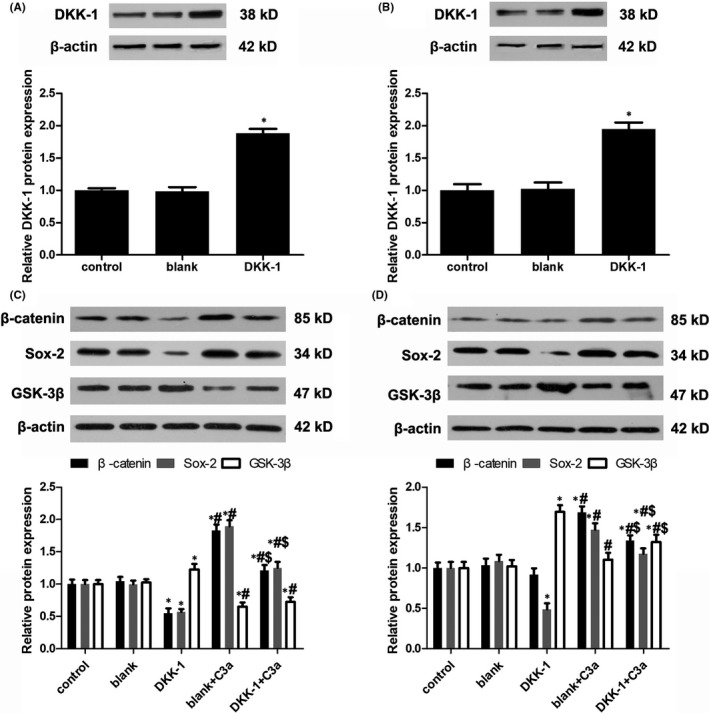
The effect of DKK‐1 overexpression on Wnt/β‐catenin pathway. (A, B) DKK‐1 plasmid transfection in A431 and SCC13 cells. n = 4. *P < 0.05 vs control. (C, D) Analysis of β‐catenin, Sox‐2 and GSK‐3β expression in A431 and SCC13 cells after DKK‐1 overexpression.*P < 0.05 vs control, #*P* < 0.05 vs DKK‐1 overexpression, $*P* < 0.05 vs DKK‐1 + C3a

To study how C3a regulates Sox‐2, we investigated the role of the Wnt/β‐catenin pathway in the self‐renewal of stem‐like cells showed in head and neck squamous cell carcinoma.^25^ C3aR expression was knocked down by C3aR siRNA transfection (Figure 4A,B). Compared with the control group, C3a exposure markedly raised β‐catenin expression and reduced GSK‐3β expression in cSCC cells (Figure 4C,D). In contrast, silencing C3aR expression inhibited β‐catenin and Sox‐2 expression and restored GSK‐3β expression (Figure 4C,D). DKK‐1, an antagonist of the Wnt and β‐catenin pathway, was overexpressed in cSCC cells after transfection with pcDNA3.1(‐)‐DKK‐1 (Figure 5A,B). DKK‐1 overexpression led to the reduction of β‐catenin and Sox‐2 expression (Figure 5C,D). These results suggest that C3a activated Sox‐2 by promoting the activity of β‐catenin.

### 3.6 Silencing C3aR impeded tumour growth in a xenograft model

**Figure 6:**
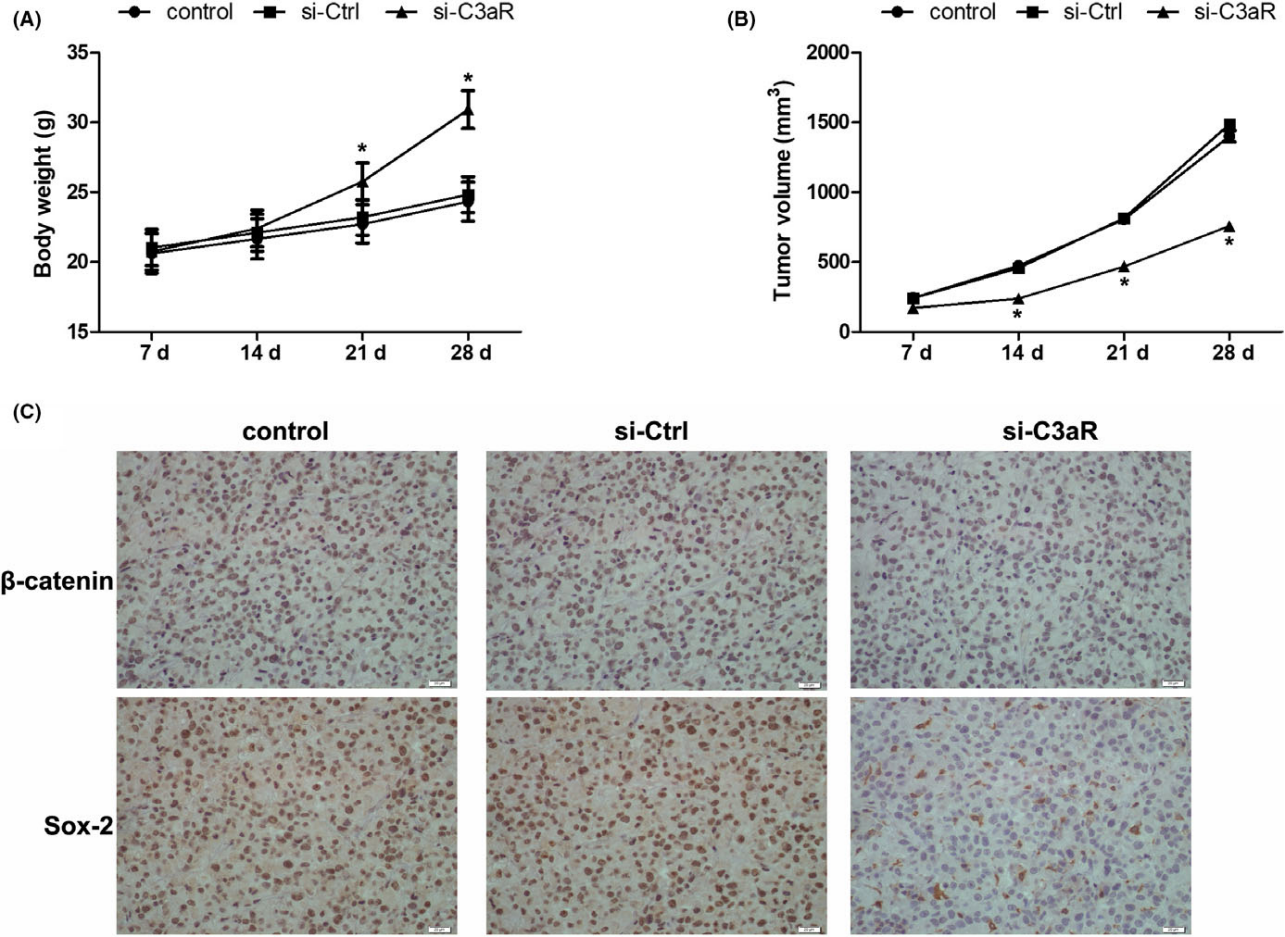
The effect of C3 on cSCC cell growth in mouse model. (A) C3aR was knocked down in A431 cells and then subcutaneously implanted into nude mice. Body weights were measured every week since the implantation. n = 5‐6. *P < 0.05 vs control. (B) Tumour volumes were measured by caliper and calculated using the formula *V* = (length × width2)/2. n = 5‐6. *P < 0.05 vs control. (C) At 4 weeks after the implantation, xenografts were harvested and the expression of β‐catenin and Sox‐2 was detected by immunohistochemistry. Scale bar = 20 μm. n = 5‐6

C3aR expression was silenced in A431 cells, which were then implanted into the backs of nude mice. Compared with the control and si‐control groups, the body weights of the mice in the C3aR siRNA group were higher at 21 and 28 days after the tumour cell injection (Figure 6A). Tumour volume also significantly decreased in the C3aR siRNA group after 14 days (Figure 6B). The results of immunohistochemistry showed that β‐catenin and Sox‐2 expression was downregulated in tumour tissues harvested from the C3aR siRNA group (Figure 6C). These results reveal that disruption of C3aR expression reduced tumour growth and β‐catenin and Sox‐2 expression in vivo.

## 4 DISCUSSION

The complement system is a central component of immunity that helps fight infection by combining innate and adaptive immune responses. In addition to pathogen elimination, complement factors contribute substantially to tissue development and regeneration. Under pathological conditions, complement factors also facilitate tumour growth and metastasis in many malignant carcinomas. C3 expression has been shown in human cancer samples and is associated with clinical features.^10^, ^26^ The present study compared C3 mRNA and protein expression in several cSCC cell lines. In line with previous studies, C3 was more highly expressed in A431, Tca8113, SCC13, HSC‐5 and HSC‐1 cells than in HaCaT keratinocytes.^15^ Hence, these cell lines may be appropriate to mimic the pathogenesis of cSCC and to explore the role of C3 in cancer cells. This study used A431 and SCC13 cells for analysis, as C3 expression is highest in these two cell lines.

When C3 encounters its convertases (C3bBb and C4b2a) and is activated following protein cleavage, complement anaphylatoxin C3a is secreted and exerts various functions in animal cells through C3aR.^27^ In this study, we found that different doses of C3a increased cell viability, promoted cyclin D1 and cyclin E expression, and stimulated cell migration in cSCC cell lines. The effect of C3a could be antagonized by blocking C3aR with its specific antagonist, which suggests that C3aR activation may be required for cSCC cell transformation. C3aR is ubiquitously expressed in many tissues like the bone marrow,^28^ brain,^29^ heart^30^ and skin.^31^ Interestingly, C3aR blockade has been shown to suppress C3 activation in mouse lungs, suggesting a dependence on C3aR in C3a production.^32^ Our results showed that the C3aR antagonist decreased cancer cell proliferation and migration, compared with the control without C3a treatment. These findings indicate that C3aR participates in complement autoactivation.

Angiogenesis mediated by VEGF is essential in solid tumour progression. In poorly differentiated cSCC, VEGF is reportedly highly expressed and correlated with p16 expression, which suggests an interaction between VEGF and p16 in the dedifferentiation of cutaneous tumours.^33^ VEGF also has been implicated in tumour immune evasion by disabling myeloid dendritic cells.^34^ Blocking neuropilin‐1, which is a co‐receptor of VEGF in cutaneous cancer stem cells, impedes cancer stemness and renewal.^35^ Our study showed that C3a treatment increased cellular VEGF expression and secretion in the supernatants of A431 and SCC13 cells. These results suggest a correlation between complement factors and cSCC dedifferentiation and stemness via VEGF upregulation. Pro‐MMP‐1 and pro‐MMP2 are critical mediators in tumourigenesis and tissue remodelling because they degrade extracellular matrix. We found that C3a treatment promoted and C3aR blockade inhibited pro‐MMP‐1 and pro‐MMP2 expression in cSCC cells.^7^, ^36^

C3a plays a pivotal role in inducing retinal stem and progenitor cell activation, independent of fibroblast growth factor.^27^ Another complement anaphylatoxin C5a and its receptor C5aR are expressed in human pluripotent stem cells, and they promote pluripotency of stem cells.^37^ Stemness factors Sox‐2, Nanog, Oct‐4 and c‐Myc control the self‐renewal and pluripotency of human pluripotent stem cells, suggesting that they are correlated with cancer stem cells.^38^ In this study, C3a exposure promoted the expression of Sox‐2, Nanog, Oct‐4, c‐Myc and CD‐44. Sox‐2 disruption ablated cell proliferation and migration induced by C3a and inhibited VEGF and MMP expression. The study provides more evidence that C3a enhances the stemness of cSCC cells.

Activation of the Wnt and β‐catenin pathway has been demonstrated in liver, pancreatic, ovarian, and colorectal and other cancers.^39^ Here, we observed that C3a treatment augmented β‐catenin expression, whereas it repressed GSK‐3β expression. DKK‐1 overexpression inhibited activation of the Wnt and β‐catenin pathway and Sox‐2 expression in cSCCs. These results underscore the mediation role of this pathway in the expression of C3a‐induced stemness factors. In a tumour xenograft model, C3aR silencing constrained tumour growth. C3aR disruption also impeded expression of Wnt‐1, β‐catenin and Sox‐2. Our findings in vivo further demonstrate that C3a and C3aR were essential for cSCC tumourigenesis and that they were correlated with the Wnt and β‐catenin pathway and Sox‐2 expression. In conclusion, C3a promoted cSCC cell proliferation and migration by modulating stemness factor expression and activation of the Wnt and β‐catenin pathway. Our study elucidated a novel correlation between complement anaphylatoxin C3a and cSCC stemness that helps to provide insights into cSCC tumorigenesis.

## CONFLICT OF INTEREST

The authors declare no conflict of interest.

## AUTHOR CONTRIBUTION

Fan and Geng designed the study; Fan, Qin and Wang performed the experiments and collected data; Qin provided help in statistical analysis; Fan wrote the manuscript; Geng edited the manuscript.
